# Günter G. Hoffmann: Infrared and Raman spectroscopy: principles and applications

**DOI:** 10.1007/s00216-023-05123-6

**Published:** 2024-01-16

**Authors:** Gerald Steiner

**Affiliations:** Klinik Und Poliklinik für Anästhesiologie und Intensivtherapie, Abt. Klinisches Sensoring Und Monitoring, Medizinische Fakultät Carl Gustav Carus, Technische Universität Dresden, Fetscher Str. 74, 01307 Dresden, Germany


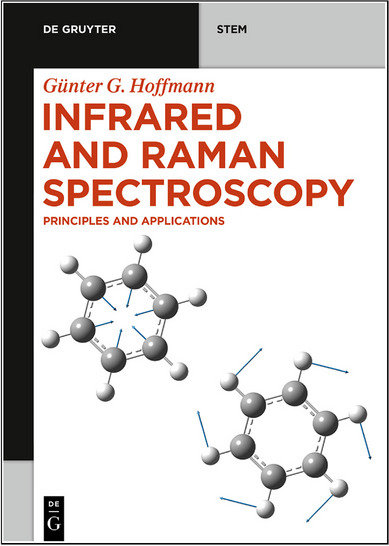


**Bibliography**

Infrared and Raman spectroscopy: principles and applications

Günter G. Hoffmann

Series: De Gruyter STEM

De Gruyter

ISBN: 9783110717549

Paperback, 431 pages

18 September 2023

## Book’s topic

Of course, the title already makes it clear what the content is. There are many books on molecular spectroscopy dealing with its two most important representatives, infrared (IR) and Raman spectroscopy. This is not surprising, as IR and Raman spectroscopy are one of the most important techniques in analytical chemistry. The availability of new lasers and detectors and the use of computationally intensive methods, including AI for spectral analysis, have significantly expanded the range of applications for IR and Raman spectroscopy. In recent years, novel applications have been developed and introduced into practice, particularly in the bio- and material sciences. Knowledge and understanding of the fundamentals are more important than ever. With this background, the book is an excellent companion for both theoretical and practical work. Like no other monograph, it combines the necessary theoretical principles, the interpretation of spectra and examples for applications.

## Contents

The book is divided into no fewer than 22 chapters, with two smaller but nice chapters devoted to the introduction. This is followed by a short description of some theoretical aspects of vibrational spectroscopy, followed by descriptions of common instruments and technical systems. By far, the largest chapter is devoted to the vibrational spectra of organic compounds, followed by natural substances and the usual observations on inorganic compounds. Many substance classes are explained in detail, in some cases also comparatively, and are rounded off with tables for band assignment. Users who are looking for an interpretation of the spectra will find a lot of data and references. However, the examples of spectrum interpretation are somewhat “Raman heavy”. There are also well-structured descriptions and explanations of special techniques with regard to application and non-linear methods of IR and Raman spectroscopy. The spectrum ranges from the spectroscopy of gases and polymers to time-resolved and non-linear Raman spectroscopic methods, which are dealt with in four separate chapters. The last four chapters again deal with special applications from industry, forensics, and art to medicine.

## Comparison with the existing literature

The book is essentially a good mixture of application-oriented basics, tables for band assignments, descriptions of special techniques, and applications. Therefore, it differs somewhat from similar books on vibrational spectroscopy. Ultimately, the book is a modernized, broad application-oriented explanation and description of IR and Raman spectroscopy. It stands between the books that are dedicated to theory in width and depth and the typical reference works on classification or spectral atlases.

## Critical assessment

The first thing you notice about the book in paperback is that the cover, including the binding, is not very sturdy. For a workbook and textbook, better quality would be highly advisable. Unfortunately, quite a few illustrations of spectra are either so small or blurred that numbers and labels in particular are illegible. In terms of content, the important fields of spectroscopic imaging techniques as well as chapters on important methods of univariate and multivariate data evaluation are missing.

## Readership recommendation

The book is recommended for newcomers to the field as well as for experienced users. This is especially true if the reader wants to explore new applications or get an overview of newer techniques. As a reference book for spectrum interpretation, i.e., band assignment, it is also recommended as an introduction or for initial orientation.

## Summary

This is more than just another book on IR and Raman spectroscopy. A good mixture of basics, the presentation of new techniques, and of course the explanations on the interpretation of spectra make the book a universal companion for IR and Raman spectroscopists. Minor compromises in the typographical design ultimately reduce the value of the book for users only slightly. A highly recommended textbook and reference work for all those who either do not own the previous standard works or would like to deal with new techniques.

